# Structural adaptability and surface activity of peptides derived from tardigrade proteins

**DOI:** 10.1002/pro.5135

**Published:** 2024-08-16

**Authors:** Giulia Giubertoni, Sarah Chagri, Pablo G. Argudo, Leon Prädel, Daria Maltseva, Alessandro Greco, Federico Caporaletti, Alberto Pavan, Ioana M. Ilie, Yong Ren, David Y.W. Ng, Mischa Bonn, Tanja Weil, Sander Woutersen

**Affiliations:** ^1^ Van 't Hoff Institute for Molecular Sciences, University of Amsterdam Amsterdam The Netherlands; ^2^ Max Planck Institute for Polymer Research Mainz Germany; ^3^ Amsterdam Center for Multiscale Modeling (ACMM) University of Amsterdam Amsterdam Netherlands; ^4^ Computational Soft Matter (CSM) University of Amsterdam Amsterdam Netherlands

**Keywords:** conformational changes, environmental tolerance, peptides, spectroscopy, tardigrade

## Abstract

Tardigrades are unique micro‐organisms with a high tolerance to desiccation. The protection of their cells against desiccation involves tardigrade‐specific proteins, which include the so‐called cytoplasmic abundant heat soluble (CAHS) proteins. As a first step towards the design of peptides capable of mimicking the cytoprotective properties of CAHS proteins, we have synthesized several model peptides with sequences selected from conserved CAHS motifs and investigated to what extent they exhibit the desiccation‐induced structural changes of the full‐length proteins. Using circular dichroism spectroscopy, two‐dimensional infrared spectroscopy, and molecular dynamics simulations, we have found that the CAHS model peptides are mostly disordered, but adopt a more α‐helical structure upon addition of 2,2,2‐trifluoroethanol, which mimics desiccation. This structural behavior is similar to that of full‐length CAHS proteins, which also adopt more ordered conformations upon desiccation. We also have investigated the surface activity of the peptides at the air/water interface, which also mimics partial desiccation. Interestingly, sum‐frequency generation spectroscopy shows that all model peptides are surface active and adopt a helical structure at the air/water interface. Our results suggest that amino acids with high helix‐forming propensities might contribute to the propensity of these peptides to adopt a helical structure when fully or partially dehydrated. Thus, the selected sequences retain part of the CAHS structural behavior upon desiccation, and might be used as a basis for the design of new synthetic peptide‐based cryoprotective materials.

## 1 INTRODUCTION

Tardigrades, also known as “water bears,” are microscopic organisms that can withstand harsh environmental conditions, such as high temperatures and desiccation (Hibshman et al., 2020; Møbjerg et al., 2011). Research into the biomolecular mechanisms behind their high‐stress tolerance has shown that certain proteins play an essential role in protecting the integrity of cellular components under extreme conditions (Boothby et al., 2017). Three novel protein families have been discovered in the past decade that are conserved among different species of tardigrades: Cytoplasmic, Secreted, and Mitochondrial Abundant Heat Soluble (CAHS, SAHS, and MAHS) proteins (Boothby et al., 2017; Hesgrove & Boothby, 2020). Although the cytoprotective mechanism of CAHS proteins is not fully understood, recent work by Boothby et al. has shown evidence that CAHS proteins under desiccation undergo a liquid‐to‐gel transition, which restricts the molecular motions of both the protein itself and the desiccation‐sensitive biological materials embedded within the gel, thereby promoting the maintenance of cell volume and the induction of reversible biostasis. This gelation is connected to a desiccation‐induced conformational change: CAHS proteins are intrinsically disordered proteins, but upon desiccation, the central domain folds into an α‐helix, whereas the external domains adopt a β‐sheet structure. Helix–helix interactions between the proteins are crucial to drive the liquid‐to‐gel transition (Sanchez‐Martinez et al., 2024).

Mimicking the structural adaptability of CAHS, SAHS, and MAHS proteins to achieve comparable biological functionality has been a long‐standing goal in the development of synthetic bionanomaterials. For the family of LEA (Late Embryogenesis Abundant) proteins, there have already been attempts to mimic their protective effect (preventing heat‐induced aggregation) by using model peptides inspired by conserved LEA motifs. A recent example is the model LEA peptide ${\left(\text{AKDGTKEKAGE}\right)}_{2}$, which can effectively inhibit heat‐induced irreversible denaturation and inactivation of lysozyme in buffer solution (Furuki et al., 2020). Similarly to such LEA‐inspired peptides, imitating some of the biological functions of CAHS proteins by using model peptides could help to develop new preservation methods for sensitive drug formulations. As a first step in this direction, we selected four peptide sequences based on conserved regions of CAHS proteins (Table 1) and studied their structural adaptability under desiccating conditions. These sequences originate from the conserved CAHS motif 1 or from motif 2 within the CAHS‐c2 region. They showcase resemblances in polar and non‐polar amino acid arrangement. Nevertheless, they also display differences in their sequence length and overall charge. This enables a comparison of how these factors affect structural adaptability in desiccating conditions. For the same reason, our investigation included an examination of the impact of sequence length by producing CAHS11aa as a truncated variant of the longer model peptide CAHS22aa. As a negative control, we chose a peptide sequence inspired by a non‐CAHS tardigrade protein, SAHS1C3. The SAHS1C3 model peptide (which we will refer to as the *control peptide*) is derived from an extracellularly active protein of the SAHS family, presenting distinct functional attributes compared to the intracellularly active CAHS proteins. Both protein families are involved in stress response and have been implicated in mediating desiccation tolerance in tardigrades (Fukuda et al., 2017). Given the differences in their conserved regions, we selected SAHS1C3 as a control, as the structural alignment of the CAHS model peptides with the SAHS1C3 control peptide showed no similarities in the amino acid pattern regarding the distribution of polar and non‐polar residues.

**TABLE 1:** CAHS model peptides and SAHS‐inspired control peptide: Names, sequence, number of amino acids and PDB code of the protein of origin.

Name	Sequence	Number	PDB
CAHS1M1	YR HQT**EAEAEK**IRR**ELEK** Q	19	J7M799 (CAHS motif 1)
CAHS1M2	**K** Q**KK** MIDVESRY**AKK** DMDRE	20	P0CU51 (CAHS motif 2)
CAHS11aa	**AE K** IR**KELEK**Q	11	P0CU45
CAHS22aa	**AE K** IR**KELEK** QHARDV**E**FR**K**SL	22	P0CU45
SAHS1C3 (control)	**K** YTEDGD**KL**VA	11	P0CU40* (except E114→ D)

*Note*: Bold letters highlight the amino acids that favor α‐helix formation (Worsfold et al., 2019).

To investigate the conformational changes of the model peptides under drying conditions, we combine circular dichroism (CD) spectroscopy with linear and two‐dimensional infrared spectroscopy (IR and 2D‐IR, respectively) and molecular dynamics (MD) simulations. CD spectroscopy can distinguish random coil, α‐helical and β‐sheet structures, as these have distinct spectral signatures (Greenfield, 2006). IR spectroscopy also reports on protein conformation, providing additional and complementary structural information. The molecular vibrations of the amide groups, in particular the amide I mode (which mainly involves the carbonyl stretching), are sensitive to the protein conformation and conformational changes (Barth, 2007; Jafari et al., 2023), and the absorption frequency of the amide I mode can be used as a secondary‐structure marker. However, the infrared absorption spectra in the amide I region are generally rather congested, limiting our ability to disentangle the underlying amide I bands in an unambiguous manner. This problem can be partially solved by using 2D‐IR spectroscopy, which has a higher spectral resolution and conformation sensitivity than conventional IR spectroscopy (Chen et al., 2023; Ganim et al., 2008; Hamm & Zanni, 2011; Hume et al., 2019; Hunt, 2009; Minnes et al., 2017). Finally, we will use sum frequency generation (SFG) spectroscopy on the amide I mode (Fu et al., 2011; Hosseinpour et al., 2020; Wang et al., 2016) to study the model peptides at the air/water interface, which has a high hydrophobicity (Van Oss et al., 2005), and hence mimics partial dehydration (Maltseva et al., 2023). Using molecular dynamics (MD) simulations, we provide insight into the structural conformations at atomistic resolution and characterize the thermodynamics of the system.

We find that all model peptides inspired by the conserved domain of CAHS proteins show a similar structural change upon drying compared to the full protein: under hydrating conditions, the peptides have an unordered structure, whereas upon dehydrating, they adopt a helical conformation. The control peptide has a more ordered structure, consisting of a bend/turn that is stabilized under drying conditions. Furthermore, sequence‐structure analysis suggests that specific patterns in the sequence, such as alternating of positive and negative charges, might play a role in stabilizing alpha‐helical conformations, as has been suggested previously (Batchelor & Paci, 2018; Biswas et al., 2024). Finally, our observations indicate that the four model peptides not only exhibit surface activity but also, consistent with bulk findings, adopt a helical structure under partially dehydrating conditions.

## 2 RESULTS

### 2.1 Experiments in solution

#### 2.1.1 Hydrating conditions

We first study the tardigrade‐inspired peptides in an aqueous solution using circular dichroism (CD) spectroscopy, (2D‐)IR spectroscopy, and molecular dynamics (MD) simulations. We combine these methods because CD spectroscopy is sensitive to helical structure (Greenfield, 2006), while IR spectroscopy can discriminate between other secondary structures (Barth, 2007; Barth & Zscherp, 2002) and is sensitive to changes in the peptide hydration. In both IR and CD spectroscopy, spectral congestion can make it difficult to disentangle the presence of specific spectral signatures that can be assigned unambiguously to distinct secondary structures. Although different data analysis methods have been proposed to solve this issue, identifying secondary structures based only on the CD spectral shapes or IR absorption frequencies obtained via fitting may not be sufficient to determine the secondary structures present. For instance, an assignment based on the absorption frequency is not always unique because of secondary effects, such as solvent interactions, that may shift the amide I vibrational frequencies, and more specific spectral signatures are required. This problem can be partially overcome by 2D‐IR spectroscopy, which has increased spectral resolution compared to conventional IR, and is specifically sensitive to secondary structure (Chen et al., 2023; Ganim et al., 2008; Giubertoni et al., 2022; Hamm & Zanni, 2011; Hume et al., 2019; Hunt, 2009; Minnes et al., 2017). This makes the combination of CD and (2D‐)IR spectroscopy a useful tool to characterize the structure of peptides in solution. The results are complemented by MD simulations, which provide detailed structural information at atomistic resolution.

**FIGURE 1:**
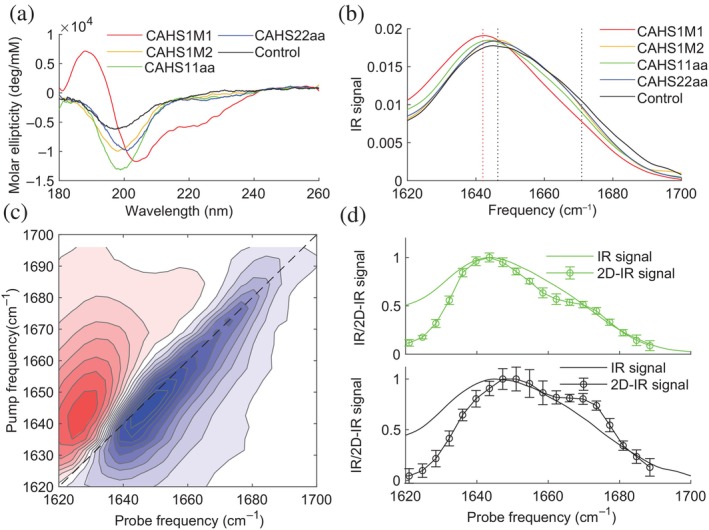
Experiments on the peptides in aqueous solution. (a) Circular dichroism (CD) spectra of the model peptides at 0% v/v TFE at a concentration of 0.1 mg/mL in buffer solution. (b) Normalized IR spectra of the model peptides at 0% v/v TFE at a concentration of ∼10 mg/mL in buffer solution (full range spectra are reported in Figure S1). (c) Isotropic 2D‐IR spectrum of CAHS11aa at a waiting time, $T_{w}=1$ ps. (d) Comparison between the IR and 2D‐IR diagonal slices of the bleach signals of the isotropic 2D‐IR spectra of CAHS11aa (top) and the control peptide (bottom).

The CD spectrum of CAHS1M1 (Figure 1a) shows a positive peak at 190 nm and two negative peaks around 210 and 225 nm, indicating an alpha‐helical structure (Greenfield, 2006), as was observed previously for the full CAHS protein (Yamaguchi et al., 2012). Interestingly, the CD spectra of CAHS1M2, CAHS11aa, and CAHS22aa differ significantly from those of CAHS1M1. These three peptides show a negative peak around 200 nm and a less intense one at 220 nm. This spectral signature can be assigned to a random‐coil (unordered) structure (Greenfield, 2006). Compared to CAHS11aa and CAHS1M2, CAHS22aa shows the minimum at a slightly higher wavelength, and a slightly more pronounced negative peak at 220 nm, suggesting a greater propensity for alpha‐helical structure (Greenfield, 2006). The SAHS‐based control peptide shows a negative peak around 200 nm, indicating a mostly unordered structure.

The IR spectrum of CAHS1M1 is centered at $1642\;{\mathrm{cm}}^{-1}$ with a shoulder around $1670\;{\mathrm{cm}}^{-1}$ (Figure 1b). The spectra of CAHS1M2, CAHS11aa, CAHS22aa, and the control have similar spectral shapes: a main band around $1648\;{\mathrm{cm}}^{-1}$ and again a shoulder around $1670\;{\mathrm{cm}}^{-1}$. In these spectra, it is challenging to disentangle the different vibrational bands that are present and uniquely identify their vibrational frequencies, which can be used to characterize the peptide secondary structures. To overcome these problems, we use two‐dimensional infrared spectroscopy (2D‐IR). Compared to the linear infrared spectra, 2D‐IR better resolves the individual vibrational bands because the 2D‐IR signal scales quadratically with the absorption cross‐section (while the conventional IR signal scales linearly with the cross‐section), leading to narrower peaks and a removal of the solvent background (Giubertoni et al., 2022; Hamm & Zanni, 2011; Hunt, 2009). In pump‐probe 2D‐IR spectroscopy, an intense, narrow‐band infrared pump pulse (with adjustable center frequency $\nu_{\text{pump}}$) resonantly excites at a specific frequency (in the present case, in the amide I band). A delayed, broad band probe pulse probes the frequency‐dependent IR‐absorption change ΔA, which results from the excitation by the pump beam. Measuring the ΔA spectra for a range of $\nu_{\text{pump}}$ values, we obtain 2‐dimensional spectra showing the pump‐induced absorption change $\Delta A\left(\nu_{\text{pump}},\nu_{\text{probe}}\right)$ as a function of the pump and probe frequencies $\nu_{\text{pump}}$ and $\nu_{\text{probe}}$ (Hamm & Zanni, 2011; Hunt, 2009). Figure 1c shows the 2D‐IR spectrum of CAHS11aa (the 2D‐IR spectra of the other peptides are shown in the SI, Figure S2). When the pump frequency is resonant with the v=0→1 frequency of the amide I mode, part of the molecules are excited to the v=1 state of this mode, causing a decrease in the absorption at the v=0→1 frequency and an increase in absorption at the v=1→2 frequency (which is at a slightly lower value than the v=0→1 frequency due to the anharmonicity of the vibrational potential), resulting in a ΔA<0 feature on the diagonal and a ΔA>0 feature slightly to the left of the diagonal. In this way, each normal mode gives rise to a +/− doublet on the diagonal of the 2D spectrum (diagonal peaks), where the peaks colored in blue represent decreases in absorption (ΔA<0) due to depletion of the amide‐I v=0 state, and the signal at lower probe frequency colored in red represents the induced absorption of the ν=1→2 transition. The diagonal peak is centered at $1640\;{\mathrm{cm}}^{-1}$, showing a shoulder at a higher frequency. Taking the diagonal slice of the 2D‐IR spectrum (Figure 1d), we can distinguish vibrational bands at 1645 and $1670\;{\mathrm{cm}}^{-1}$. Similarly, we better resolve the vibrational bands underlying the IR spectrum of the control peptide (Figure 1d, lower panel). Interestingly, the band at $1675\;{\mathrm{cm}}^{-1}$ is more pronounced in the control peptide than in the CAHS11aa (which is not clearly visible in the IR spectra). A complete comparison between the diagonal slices of all model peptides is shown in the SI (Figure S2). These two bands can be assigned to random coil ($1648\;{\mathrm{cm}}^{-1}$) and bend/turn ($1675\;{\mathrm{cm}}^{-1}$) structures (Barth, 2007). The IR spectrum of CAHS1M1 shows a main band at a lower frequency than the other peptides, which we attribute to an increased alpha‐helical content based on the CD results. These results indicate that three of our chosen model peptides favor a mostly unordered structure in aqueous solution with a small propensity to adopt a helical structure, and one (CAHS1M1) is mostly helical in hydrating conditions. Although the control peptide also prefers random coil structure, the relatively higher intensity of the bend/turn peak indicates a stronger propensity to form a bend/turn structure than the other model peptides.

**FIGURE 2:**
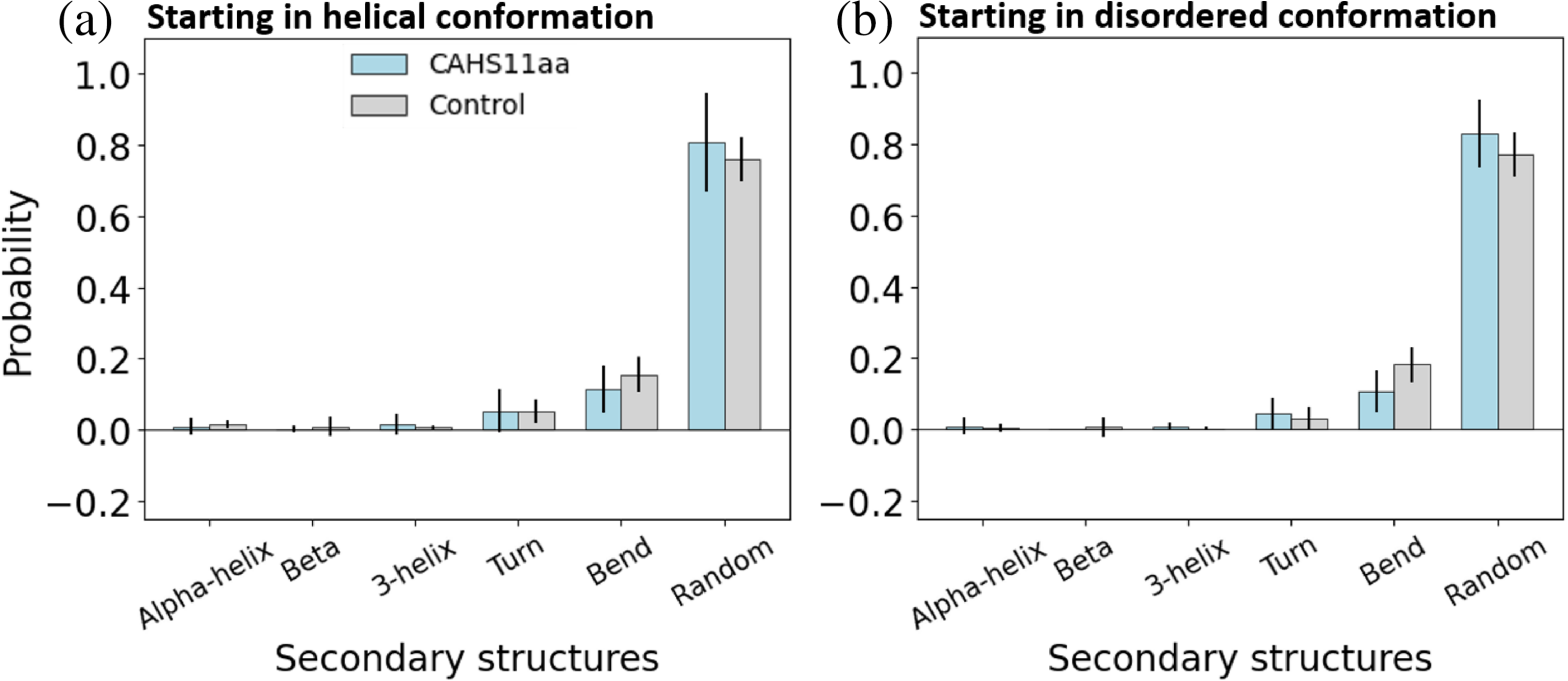
Molecular dynamics (MD) simulations of the peptides in aqueous solution. Secondary structure analysis in hydrating conditions when averaged over the contributions of all residues. Two different sets of simulations are analyzed: Started from helical structures (a) and started from randomly generated conformations (b). The error bars represent the standard error of the mean calculated as the standard deviation of the average values over the independent runs.

To obtain further information on the conformational stability of the peptides in hydrating conditions, we carried out molecular dynamics simulations of CAHS11aa and the control peptide in water by performing 10 independent unbiased simulations for each system in water (cumulating 20 μs). Since the three‐dimensional structures of the peptides are unknown, the starting conformations were generated using two different protocols: five initial conformations per system starting from helical conformations (Jumper et al., 2021) (*protocol A*), and five randomly generated conformations (Ilie et al., 2022; Ilie & Caflisch, 2018) (*protocol B*). Figure 2 shows the structural analysis for the two peptides, obtained using both protocols for generating the initial conformation (for further details, see Section 4 and Figures S4, S5 and S6). We observe that both peptides adopt mostly non‐helical structure and that the control peptide more frequently forms bend/turn structures independently of the starting configuration (Figure 2, S4 and S5) in agreement with the 2D‐IR results. Interestingly, the MD simulations also revealed that the central stretch 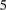
DGD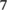 of the control peptide attains bend/turn conformations over 50% of the simulation time, whereas this percentage is significantly less for the residues in the CAHS11aa peptide (Figure S6).

#### 2.1.2 Dehydrating condition

**FIGURE 3:**
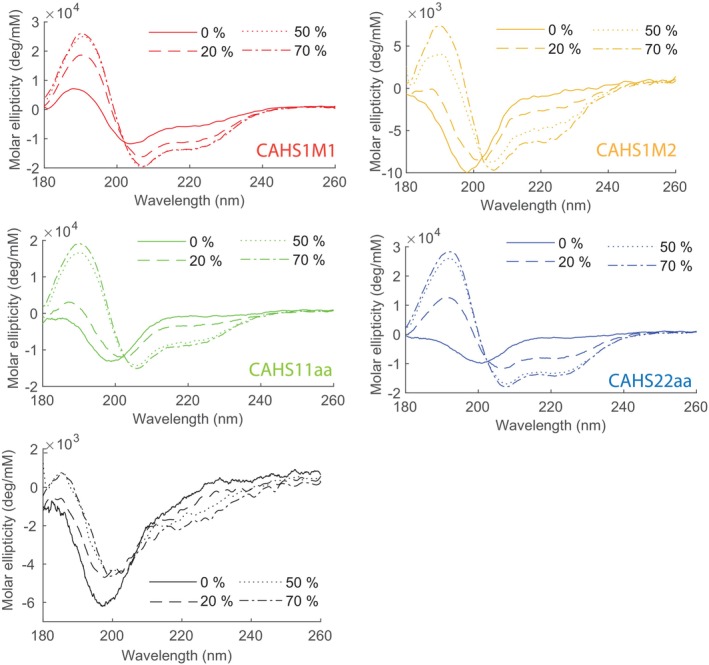
Experiments on the peptides in dehydrating circumstances. Circular dichroism spectra of the model peptides dissolved in buffered water/TFE mixtures at a concentration of 0.1 mg/mL, with increasing TFE fraction to mimic dehydration.

We study conformational changes induced by water‐deficient conditions by performing a titration with the desolvating agent TFE (2,2,2‐trifluoroethanol) in order to mimic desiccation (Wang et al., 2023). First, we measure the CD spectra of peptides by increasing the TFE content from 0 to 20, 50, and 70% v/v. These experiments showcase the specific properties of the CAHS model peptides, which are comparable in terms of folding as the above‐mentioned LEA model peptides (Shimizu et al., 2010). As the level of the desolvating agent, TFE was increased from 20%, 50% to 70%, the CD signals at 190 nm and 208 nm increased significantly, indicating an increase in α‐helical content of the peptide secondary structure of CAHS model peptides (Figure 3). In contrast, the control peptide showed no significant changes in secondary structure, even at 70% v/v TFE content (bottom in Figure 3).

**FIGURE 4:**
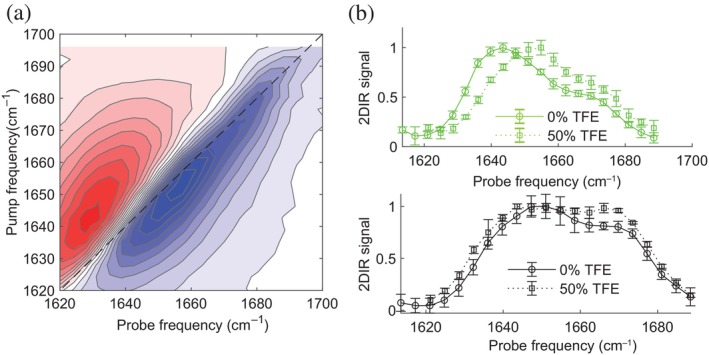
Experiments on the peptides in dehydrating circumstances. (a) 2D‐IR spectrum of CAHS11aa at 50% v/v TFE concentration. (b) Comparison of the 2D‐IR diagonal slices of the bleach signals of the isotropic 2D‐IR spectra of CAHS11aa (top) and control (bottom) peptides at 0 and 50% v/v TFE concentration.

To obtain more structural information, we record 2D‐IR spectra at 50% v/v TFE content for CAHS11aa and the control peptide (conventional IR spectra are shown in the SI, Figure S3). Figure 4a shows the 2D‐IR spectrum of CAHS11aa at 50% v/v TFE. Compared to the 2D‐IR spectrum of Figure 1c, the main peak is shifted to a higher frequency. In the diagonal‐slice spectrum, the main band also shifts to a higher frequency (from 1645 to $1655\;{\mathrm{cm}}^{-1}$) in the presence of TFE. A similar shift was observed previously for LEA protein, where the absorption peak blueshifts from 1648 to $1660\;{\mathrm{cm}}^{-1}$ upon adding TFE because of the formation of the helical structure (Koubaa et al., 2019). Based on these previous results, we assign the band at $1655\;{\mathrm{cm}}^{-1}$ to the alpha‐helical structure, its increase with increasing TFE fraction confirming the increase in alpha‐helix content observed in the CD spectra. Figure 4b shows the diagonal slices of the control peptide at 0% and 50% TFE (for which hardly any change occurs in the CD spectrum upon increasing the TFE concentration, see Figure 3). The diagonal slices show that the band at $1670\;{\mathrm{cm}}^{-1}$ increases in intensity, suggesting that drying increases the propensity of the control peptide to form a bend/turn structure.

**FIGURE 5:**
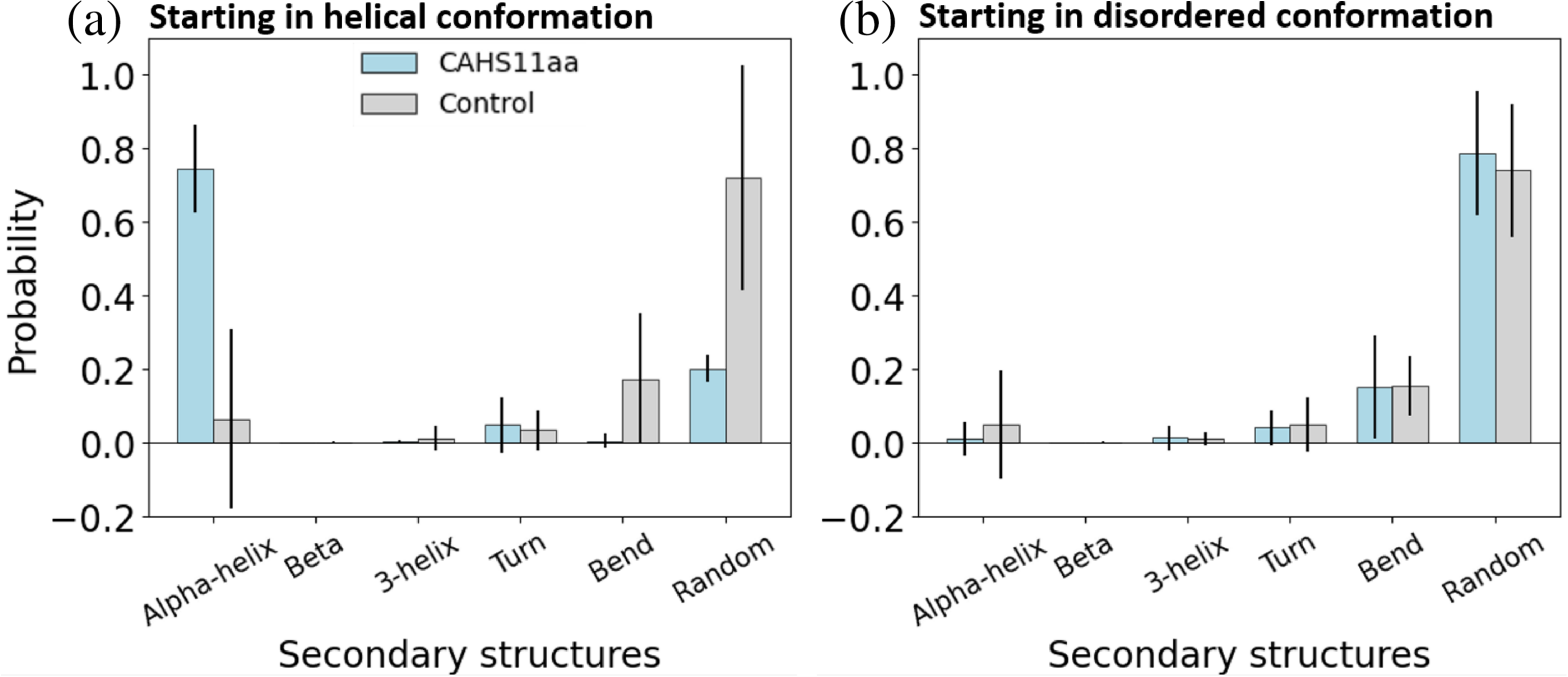
Secondary structure analysis in 70% TFE when averaged over the contributions of all residues. Two different sets of simulations are analyzed: Started from helical structures (a) and started from randomly generated conformations (b). The error bars represent the standard error of the mean calculated as the standard deviation of the average values over the independent runs.

The absence of the formation of helical structure in the case of the control peptide in the presence of TFE is also observed in our MD simulations. The structural analysis in 70% v/v TFE, extracted from the molecular dynamics simulations (Figure 5), reveals that the control peptide attains mainly coil and bend/turn‐like conformations independently from the starting conformation. A similar structural analysis was also carried out for CAHS11aa in 70% v/v TFE. However, we observed interestingly that the structural behavior of CAHS11aa in the presence of TFE showed a strong dependency on the protocol used for generating the starting structure. Contrary to the control peptide, CAHS11aa preserves to a large extent its helical conformation in 70% v/v TFE when started from the extended helix generated using protocol A (Figures 5 and S7C); it only occasionally attains helical conformations when started from random conformation, generated using protocol B (Figures 5 and S7D). Such strong dependency on the initial structure suggests that the CAHS11aa needs to overcome a free‐energy barrier in order to become an extended helix (see the next section).

#### 2.1.3 Conformational preferences and thermodynamic analysis

**FIGURE 6:**
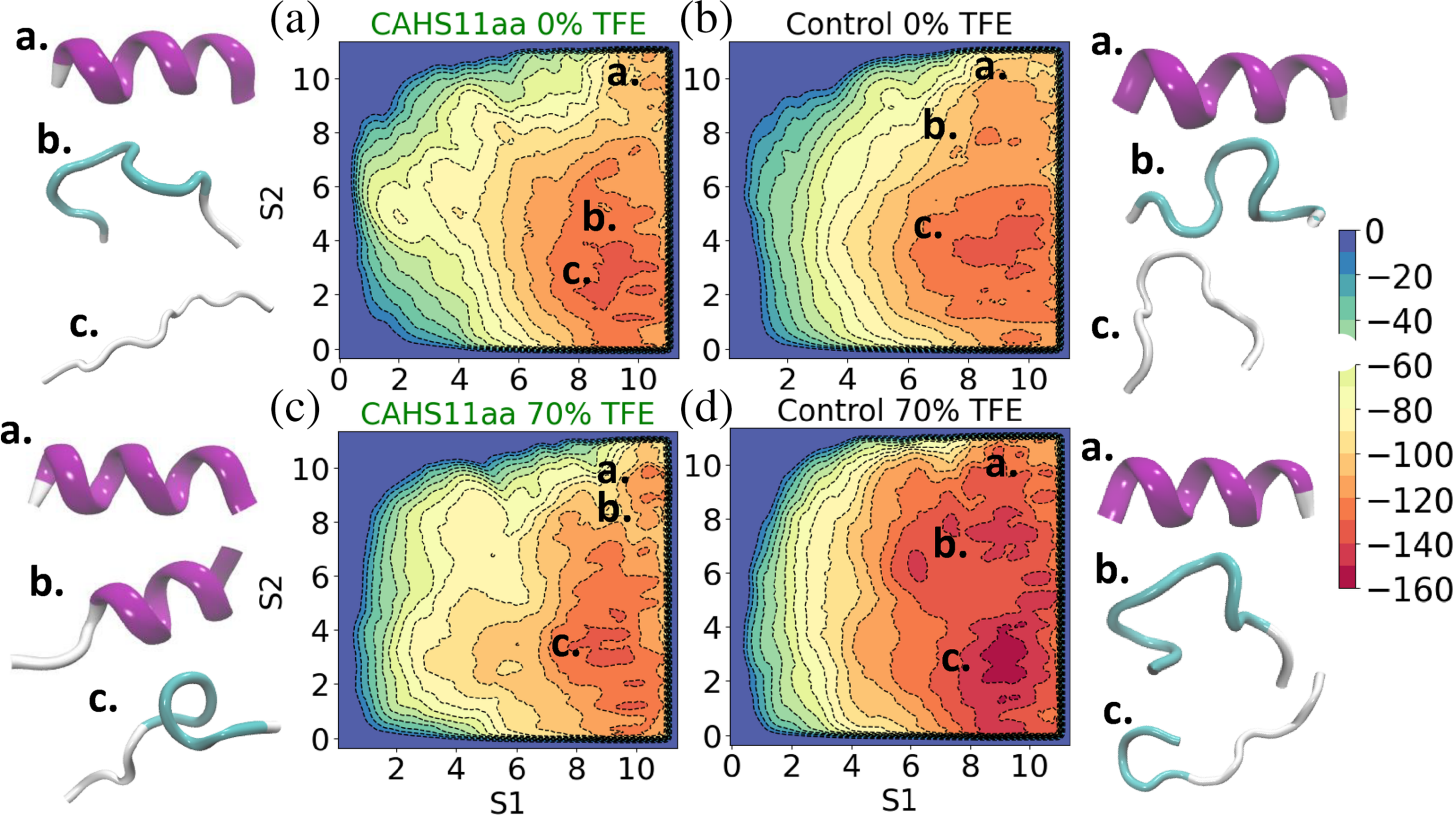
Simulations of the peptides in normal and dehydrating circumstances. Free energy surface as a function of the collective variables S1 and S2 for the CAHS11aa and the control peptide in hydrating and under drying conditions (0 and 70% v/v TFE, respectively). The snapshots show representative, yet not unique, conformations of the peptides in the highlighted regions. The free energy scale is in kJ ${\mathrm{mol}}^{-1}$.

To quantitatively characterize the conformational preferences under hydrating and drying conditions, we performed metadynamics simulations of the CAHS11aa and the control peptide in water and 70% v/v TFE. First, we analyzed the free energy landscapes of the peptides as a function of the collective variables $S_{1}$ and $S_{2}$ (see the Section 4 for their definition), which measure the deviations from an extended helical conformation used as a reference (snapshots “a” in the upper right corners in Figure 6). All metadynamics simulations are started from extended helical conformations, α‐conformations (snapshots a. in the upper right corners in Figure 6). In Figure 6, we plot the obtained profiles, showing rugged free energy landscapes populated by multiple local minima surrounded by free energy barriers, which are common for intrinsically disordered polypeptides (de Raffele & Ilie, 2024; Ilie & Caflisch, 2019). Each minimum corresponds to a different conformational state. We observe that α‐conformations are disfavored in hydrating conditions (no minimas in Figure 6a,b), indicating that the peptides do not attain stable α‐conformations in water, in line with the previous MD and experimental results. Contrary to the hydrating conditions, we observe that in 70% v/v TFE, the peptides sample helical conformations in a narrow regime, as evidenced by the relatively narrow minima (Figure 6c,d for S1, S2∈[9.5, 11]). Notably, the CAHS11aa peptide in 70% v/v TFE has the highest propensity to sample extended helical conformations, with the free energy difference between α and non‐α‐structures $\Delta G_{\alpha ,1-\alpha }=14.1$ kJ/mol. Our results show that CAHS11aa in 0% v/v TFE, and the control peptide in both 0 and 70% v/v TFE have comparable disfavored propensities to attain extended helical conformations (Figure S9) with $\Delta G_{\alpha ,1-\alpha }=23.5$ kJ/mol, $\Delta G_{\alpha ,1-\alpha }=24.1$ kJ/mol and $\Delta G_{\alpha ,1-\alpha }=25.2$ kJ/mol, respectively. The simulations support the results from the unbiased simulations and from the CD and 2D‐IR experiments, which show a higher propensity for CAHS11aa than for the control peptide to attain a helical structure.

### 2.2 Conformation at the air/water interface

**FIGURE 7:**
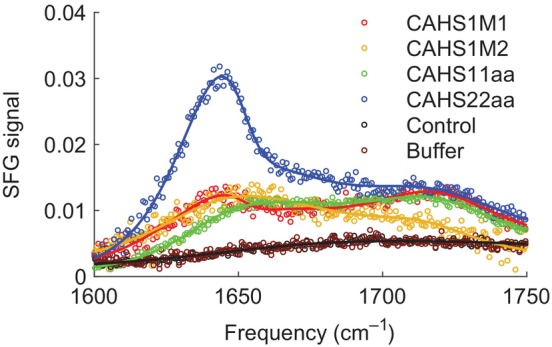
Experiments on the peptides at the water/air interface. SSP amide I SFG spectra (open symbols) along with fits (solid lines) for the five model peptides and the buffer used at the air/water interface.

Finally, we characterize the behavior of the peptides at the air/water interface. Due to its hydrophobic nature, this interface mimics desiccation (Van Oss et al., 2005). In order to characterize the peptide structure, we conduct amide I Sum‐Frequency Generation (SFG) spectroscopy on the peptides. SFG provides information about the structure and the orientation of interfacial proteins, without interference from the ones present in the aqueous media and a more hydrated environment (Hosseinpour et al., 2020). In a biological environment, peptides will be exposed to hydrophobic regions of, for instance, proteins and lipids. As such, an understanding of the behavior of peptides in a hydrophobic environment complements the insights gained from studies of peptides in solution. Primarily arising from the carbonyl stretch of the amide bond, due to excitonic coupling, the amide I vibrational response is sensitive to the peptide conformation (Barth & Zscherp, 2002). Moreover, employing an SSP (s‐polarized SFG, s‐polarized VIS, p‐polarized IR) polarization, the obtained spectra solely present the backbone amide I band, effectively eliminating side chain contributions and allowing an unobstructed analysis of the peptide's orientation (Lu et al., 2018). It is important to note that the SFG signal intensity is determined not only by the number of proteins on the surface, but also by their orientation at the air/water interface. Specifically, the SSP geometry is sensitive to functional groups positioned perpendicular to the sample surface (Clarke et al., 2005). Figure 7 shows the SFG spectra together with fitted curves obtained from a spectral analysis (see the SI, where the fit parameters can also be found). Since only ordered structures are visible in the SFG spectra, the presence of the amide I mode implies that the CAHS peptides occupy the interface and form a well‐aligned layer at the air/water interface. Furthermore, consistent with the CD and 2D‐IR results under drying conditions, the peptides predominantly adopt an α‐helical conformation. This is evidenced by a peak at ∼1645 cm^−1^, characteristic of α‐helical structures (Lu et al., 2018; Lutz et al., 2015; Lutz et al., 2017; Xiao et al., 2018). The mode at ∼1720 cm^−1^, typically attributed to the carbonyl vibrations of amino acid side chains, (Dreier et al., 2019) can be assigned to protonated glutamic acid and aspartic acid side chains of the peptides (Roeters et al., 2021). Both amino acids can assume a protonated state within the peptide when present in a neutral pH environment (Fersht, 1999; Honig & Hubbell, 1984). In contrast, the control peptide does not display interfacial assembly and organization at the measured concentration, yielding a spectrum indistinguishable from that of the buffer subphase. Of interest is the enhanced SFG activity of CAHS22aa. As mentioned earlier, the intensity of the SSP SFG signal at the air/water interface is determined by the number of peptides on the surface and their degree of collective order. Furthermore, CAHS22aa is the only peptide with aromatic residues, which could enhance SFG signals through a larger Raman response. All in all, it seems that the increased signal for CAHS22aa can be traced in part to the greater surface propensity of CAHS22aa with respect to the other peptides CAHS11aa and CAHS1M1, as indicated by their surface pressure values in Figure S4. This effect is likely compounded by enhanced interfacial inversion‐symmetry breaking due to π‐π interactions between the peptides. Finally, a broad low‐intensity spectral feature is observed over the entire range of the amide I region corresponding to the H‐O‐H bending mode of the water.

## 3 DISCUSSION AND CONCLUSION

From the joint CD, (2D‐)IR, and molecular dynamics simulations, we conclude that in hydrating conditions the structure of three model peptides (CAHS1M2, CAHS11aa, and CAHS22aa) and the control is mostly disordered, with different preferences for adopting a transient helical conformation. Interestingly, CAHS1M1 adopts a mainly helical configuration already at 0% TFE. Upon mimicking desiccation (either by adding TFE or by exposing the peptides to a water/air interface and characterized by SFG), all model peptides adopt a more helical conformation, similar to what is observed for the entire protein (Biswas et al., 2024; Sanchez‐Martinez et al., 2024). The control peptide, however, does not show any tendency to form a helical structure and thus SFG signal, only a slightly higher propensity towards bend‐like structures. Furthermore, it does not show any surface activity, as opposed to the other peptides.

The specific molecular origin of the difference in the secondary structure formation and overall physicochemical behavior between the CAHS‐inspired peptides and the SAHS‐inspired control peptide remains elusive. However, there are similarities in the amino acid sequences of the CAHS‐inspired peptides that are noteworthy: first, the CAHS‐inspired peptides are richer in polar residues. These are arranged in alternating sequences of positively charged (K and R) and negatively charged amino acids (E and D). This pattern resembles the polyampholytic sequences found in both natural and synthetic helical polypeptides (Batchelor & Paci, 2018). Second, the CAHS‐inspired peptides contain a higher number of specific amino acids (M, A, L, E, and K) in their sequences compared to the control peptide. These particular amino acids are known to promote helical structures in peptide secondary structures (Pace & Scholtz, 1998). Among the model peptides, CAHS1M1 stands out as it has the highest occurrence of these amino acids: 10 out of 19 amino acids in CAHS1M1 are MALEK amino acids, occurring in two consecutive sequences of 6 (EAEAEK) and 4 (ELEK) residues each. This feature of CAHS1M1 could be a factor in its higher propensity to form a more helical secondary structure, even in the absence of desiccation‐mimicking agents like TFE.

In conclusion, our results show that in dehydrating conditions CAHS model peptides undergo structural changes similar to the full‐length proteins, known to be related to dehydration protection in tardigrades. Based on these results, we speculate that polar residues might play a role in the coil‐to‐helical transition of peptides inspired by CAHS. Thus, this work offers promising sequences for the design of new synthetic peptide‐based systems capable of mimicking the cytoprotective properties of dehydrated biologics and a generic rule for selecting specific sequences to use as a template. Our goal in the near future is to test whether novel synthetic model systems based on the CAHS model peptides presented here can mimic the cytoprotective properties of the naturally occurring tardigrade proteins (Hesgrove & Boothby, 2020).

## 4 MATERIALS AND METHODS

### 4.1 Peptide synthesis

CAHS11aa was synthesized using the Fmoc solid phase peptide synthesis strategy by Merrifield, synthesizing the peptide from C to N‐terminus in a microwave‐assisted peptide synthesizer. Fmoc–Gln (Trt) preloaded Wang resin (100–200 mesh) (0.1 mmol) was swollen in DMF for 1 h before use. First, the Fmoc group was cleaved by two deprotection steps using 20% v/v piperidine in DMF (3 mL) for 2 and 5 min at 75 Â°C. Fmoc‐Lys(Boc), Fmoc‐Glu(OtBu), Fmoc‐Leu, Fmoc‐Arg(Pbf), Fmoc‐Ile, and Fmoc‐Ala (5 equiv in 2.5 mL) were coupled for 20 min using DIC (5 equiv in 1 mL) and Oxyma (10 equiv) in 0.5 mL DMF The peptide was purified by HPLC using the Atlantis T3 column at a 10 mL/min flow rate. The gradient started at a 95:5 ratio of water:ACN (+0.1% TFA) and was kept constant for 1 min, after which the ACN content was increased to 30% in 20 min. The retention time of CAHS11aa was 3.0 min. The peptide was received in a yield of 7.1% (21 mg). CAHS22aa was synthesized using the Fmoc solid phase peptide synthesis strategy by Merrifield, synthesizing the peptide from C to N‐terminus in a microwave‐assisted peptide synthesizer. Fmoc–Leu preloaded Wang resin (100–200 mesh) (0.1 mmol) was swollen in DMF for 1 h before use. First, the Fmoc group was cleaved by two deprotection steps using 20% v/v piperidine in DMF (3 mL) for 2 and 5 min at 75 Â°C. Fmoc‐Lys(Boc), Fmoc‐Glu(OtBu), Fmoc‐Leu, Fmoc‐Arg(Pbf), Fmoc‐Ile, Fmoc‐Ala, Fmoc‐His(Trt), Fmoc‐Asp(OtBu), Fmoc‐Val, Fmoc‐Phe, and Fmoc‐Ser(tBu) (5 equiv in 2.5 mL) were coupled for 20 min using DIC (5 equiv in 1 mL) and Oxyma (10 equiv) in 0.5 mL DMF. The peptide was purified by HPLC using the Atlantis T3 column at a 10 mL/min flowrate. The gradient started at a 95:5 ratio of water:ACN (+0.1% TFA) and was kept constant for 1 min, after which the ACN content was increased to 30% in 20 min. The retention time of CAHS22aa was 1.5 min. The peptide was received in a yield of 2.0% (1.5 mg). CAHS1M1, CAHS1M2, and SAHS1C3 (control) were purchased via custom peptide synthesis from Jiangsu GenScript Biotech Co., Ltd. and received at ≥95% purity.

### 4.2 Circular dichroism spectroscopy

CD spectra were recorded on a JASCO J‐1500 spectrometer in a 0.1 cm High Precision Cell by HellmaAnalytics. The recorded data were processed in Spectra Analysis by JASCO and OriginPro9. For the preparation of the peptide solutions, each model peptide was dissolved in phosphate buffer (PB, 10 mM, pH 7.4) or a mixture of phosphate buffer and TFE (20, 50, and 70, % v/v respectively) to achieve a peptide concentration of 0.1 mg/mL in each sample. The samples were subsequently measured, and the spectra were recorded at wavelengths from 260 to 180 nm with a bandwidth of 1 nm, data pitch of 0.2 nm and scanning speed of 5 nm/min. Spectra were measured three times and accumulated.

### 4.3 Infrared and two‐dimensional infrared spectroscopy

A Perkin‐Elmer Spectrum‐Two FTIR spectrometer (resolution 2 cm^−1^) was used to measure the FTIR spectra of the background and peptide solutions. A detailed description of the setup used to measure the 2D‐IR spectra (Huerta‐Viga et al., 2010) PHSpectroscopy. Briefly, pulses of wavelength 800 nm and with a 40 femtosecond duration are generated by using a Ti:sapphire oscillator, and further amplified by using a Ti:sapphire regenerative amplifier to obtain 800 nm pulses at 1 kHz repetition rate. These pulses are then converted in an optical parametric amplifier to obtain mid‐IR pulses (∼20 μJ, ∼6100 nm) with a full width at half max (FWHM) of 150 cm^−1^. The beam is then split into a probe and reference beam (5% each), and a pump beam (90%) that is aligned using a Fabry‐Pérot interferometer. The pump and probe beams are overlapped in the sample in an ∼250‐μm focus. The transmitted spectra of the probe (T) and reference ($T_{0}$) beams with the pump on and off are then recorded after dispersion using an Oriel MS260i spectrograph (Newport, Irvine, CA) onto a 32‐pixel mercury cadmium telluride (MCT) array. The probe spectrum is normalized to the reference spectrum to compensate for pulse‐to‐pulse energy fluctuations. The 2D‐IR signal is obtained by subtracting the probe absorption in the presence and absence of the pump pulse. Parallel and perpendicular 2D‐IR spectra are recorded by rotating the pump beam at a 45° angle with respect to the probe beam and selecting the probe beam component that is either perpendicular or parallel to the pump beam using a polarizer after the sample. To minimize pump‐scattering contributions, we measured the average of two photoelastic‐modulator‐induced pump delays, such that the time delay between the scattered pump beam and the probe beam differs by 1/2 optical cycle in one delay with respect to the other. For all IR and 2D‐IR measurements at 0% TFE, peptides were dissolved in phosphate buffer (10 mM, pH 7.4) at a concentration of 10 mg/mL, and the pH was adjusted to neutral by adding an adequate amount of NaOD. For all IR and 2D‐IR measurements in the presence of TFE (deuterated, Sigma Aldrich), an adequate amount of TFE was added to the buffer solution with an adequate amount of NaOD in order to obtain a solution with 50% v/v fraction of TFE reaching a peptide concentration of 10 mg/mL. Such TFE volume fraction was chosen to minimize the dilution of the sample.

### 4.4 Sum frequency generation spectroscopy

The SFG laser system consists of a seed and pump laser, a regenerative amplifier, and an optical parametric amplifier (OPA). Pulses with a 40 femtosecond duration are generated by using a Ti:sapphire seed laser (Mai Tai, Spectra Physics), stretched in time, and the selected ones are directed into a regenerative amplifier (Spitfire Ace, Spectra Physics). The Ti:Sapphire crystal in the amplifier is pumped with an Nd:YLF laser (Empower 45, Spectra Physics). After amplification, the selected pulses were guided through a compressor, which effectively restored them to their original 40 femtosecond duration. The output beam (1 kHz repetition rate, 800 nm wavelength) is split into two paths. The first path goes to an etalon (SLS Optics) to produce a spectrally narrow visible beam (FWHM ∼15 cm^−1^). The second path is used to pump an OPA (TOPAS‐C, Light Conversion), in which the signal and idler beams are generated. The broadband infrared beam is generated by difference frequency generation from these beams. Finally, the SFG signal is detected by an electron‐multiplied charge‐coupled device camera (Newton EMCCD 971P‐BV, Andor Technology). The polarization state of the SFG, Vis, and IR beams is controlled by polarizers and half‐wave plates. In all experiments, SSP (s‐polarized SFG, s‐polarized VIS, p‐polarized IR) polarization combination was used (where p/s denotes the polarization parallel/perpendicular to the plane of incidence defined by the direction of incidence of the beam and the perpendicular to the interface). Each experiment was performed using PBS buffer as the subphase under neutral pH conditions, a final peptide concentration of 0.09 mg/mL after injection, and a minimum time of 40 min. The samples were constantly rotated at 6 rpm, kept in a closed box, and subjected to a continuous flow of nitrogen to prevent absorption of infrared radiation by air. To process each SFG spectrum, the background‐corrected sample spectrum was divided by the background‐corrected reference spectrum to correct for the spectral shape of the IR beam. The background was recorded by blocking the IR beam. A z‐cut quartz crystal was used as the reference. Each sample spectrum was recorded for 10 min, while the reference spectrum was for 10 seconds. The SFG spectra were fitted with Lorentzian peak shapes, following the procedure described elsewhere (Hosseinpour et al., 2020).

### 4.5 Molecular dynamics simulations

Ten different initial conformations were generated for the CAHS11aa and the control SAHS1C3 peptide. Five peptides were generated using Alphafold2 (Jumper et al., 2021), which predicted helical conformations for CAHS11aa with high confidence, and disordered structures for SAHS1C3. Five peptides per system were reconstructed using Monte Carlo sampling. More precisely, the residues were built initially in an excluded volume‐obeying manner using CAMPARIv3 (http://campari.sourceforge.net/V3http://campari.sourceforge.net) and then subjected to 1,000,000 elementary Monte Carlo steps (Ilie et al., 2022; Ilie & Caflisch, 2018). This procedure randomizes dihedral angles hierarchically and guarantees that there is no spurious correlation between the starting models. All simulations were carried out using the GROMACS 2019.4 simulation package (Berendsen et al., 1995; Hess et al., 2008) and the CHARMM36m force field (Huang et al., 2016). Four simulation systems were prepared consisting of a peptide (CAHS11aa or SAHS1C3) in 0 and 70 %v/v TFE. Since the CD signal did not show any additional difference when increasing the TFE concentration from 50 %v/v to 70 %v/v, we chose to perform our simulations at the highest TFE concentration only. Ten independent simulations were carried out for each system for a total sampling time of 20 μs. The N‐ and C‐termini of all fragments were capped with ACE and NME groups, respectively. For the systems with TFE, the molecules were added around the peptide prior to solvation. Each complex was then solvated in a cubic box (edge length of 6.9 nm) with TIP3P water molecules (Jorgensen et al., 1983) to which 150 mM NaCl was added, including neutralizing counterions. Following the steepest descent minimization, the systems were equilibrated under constant pressure for 5 ns, with position restraints applied on the heavy atoms of the proteins. The temperature and pressure were maintained constant at 300 K and 1 atm, respectively, by using the modified Berendsen thermostat (0.1 ps coupling) (Bussi et al., 2007) and barostat (2 ps coupling) (Berendsen et al., 1984). The production simulations were performed in the NVT ensemble in the absence of restraints. Given that the initial conformations of the peptides have been artificially generated, the first 500 ns of the NVT runs were considered as equilibration and only the subsequent 500 ns were used for the analysis. The short‐range interactions were cut off beyond a distance of 1.2 nm, and the potential smoothly decays to zero using the Verlet cutoff scheme. Periodic boundary conditions were used, and the Particle Mesh Ewald (PME) technique (Darden et al., 1993) was employed (cubic interpolation order, real space cutoff of 1.2 nm and grid spacing of 0.16 nm) to compute the long‐range electrostatic interactions. Bond lengths were constrained using a fourth‐order LINCS algorithm with 2 iterations (Hess et al., 1997). In all simulations, the time‐step was fixed to 2 fs, and the snapshots were saved every 50 ps.

### 4.6 Metadynamics simulations

To characterize the conformational landscape of the peptides, we performed metadynamics simulations using Plumed 2.70 (Tribello et al., 2014). This method enables the construction of a history‐dependent bias potential in the space of selected collective variables (CVs). The purpose of the bias is to push away the system from local minima and assist it to visit new regions of the landscape by accelerating conformational transitions. Here, two Alphabeta collective variables, $S_{1}$ and $S_{2}$ (Ilie et al., 2018; Tribello et al., 2014), were used. These are linear combinations of the deviations of the dihedral angles, $\phi_{i}$ and $\psi_{i}$ from their reference values $\phi_{\mathrm{ref}}$ and $\psi_{\mathrm{ref}}$, respectively. Hence, $S_{1}=\frac{1}{2}\sum_{i=1}^{N}\left[1+\cos\left(\phi_{i}-\phi {\mathrm{ref}}_{i}\right)\right]$ and $S_{2}=\frac{1}{2}\sum_{i=1}^{N}\left[1+\cos\left(\psi_{i}-\psi {\mathrm{ref}}_{i}\right)\right]$ with N the number of amino acids in the peptide. The reference structures correspond to full‐length α‐helical conformations of the peptides. For CAHS11aa, the reference torsion angles were calculated from the Alphafold2 (Jumper et al., 2021) structure, which predicted an extended helical conformation with high confidence. As the SAHS1C3 peptide does not exhibit extended helical conformations in solution nor is predicted to attain helical conformations, the chosen reference torsion angles are the same as for CAHS11aa. The simulated starting peptide was obtained by mutating the residues in the CAHS11aa sequence to SAHS1C3 amino acids. Four different metadynamics simulations were performed for CAHS11aa and control peptides starting from the extended helical conformations, two of which in water and two in solution with 7 M TFE. Each simulation run for of 1 μs. Gaussians were deposited every 1 ps, with a height of ω = 0.15 kJ/mol and a width of σ = 0.1.

## AUTHOR CONTRIBUTIONS

**Giulia Giubertoni:** Conceptualization; methodology; data curation; formal analysis; investigation; visualization; writing – review and editing; writing – original draft; validation. **Sarah Chagri:** Conceptualization; methodology; data curation; investigation; validation; formal analysis; writing – original draft; writing – review and editing. **Pablo G. Argudo:** Writing – original draft; writing – review and editing; investigation; formal analysis; methodology; conceptualization; validation; data curation; visualization. **Leon Prädel:** Methodology; investigation; data curation; formal analysis; writing – review and editing; validation. **Daria Maltseva:** Conceptualization; investigation; supervision; data curation; formal analysis; methodology; writing – review and editing. **Alessandro Greco:** Methodology; investigation; writing – review and editing; formal analysis; data curation. **Federico Caporaletti:** Writing – review and editing; investigation; data curation; methodology; formal analysis. **Alberto Pavan:** Investigation; methodology; validation; visualization; writing – review and editing. **Ioana M. Ilie:** Conceptualization; methodology; supervision; funding acquisition; resources; writing – original draft; writing – review and editing. **Yong Ren:** Conceptualization; methodology; supervision; validation; formal analysis; writing – original draft; writing – review and editing. **David Y.W. Ng:** Conceptualization; methodology; formal analysis; supervision; project administration; resources; writing – original draft; writing – review and editing. **Mischa Bonn:** Conceptualization; methodology; funding acquisition; project administration; resources; supervision; writing – review and editing; writing – original draft. **Tanja Weil:** Conceptualization; methodology; supervision; funding acquisition; project administration; resources; writing – review and editing. **Sander Woutersen:** Conceptualization; writing – original draft; writing – review and editing; methodology; investigation; project administration; resources.

## FUNDING INFORMATION

NWO, VI.Veni.212.240.

## CONFLICT OF INTEREST STATEMENT

The authors declare no conflict of interest.

## Supporting information


**Data S1.** Supporting information.
