# Supplementary material for: Structural adaptability and surface activity of peptides derived from tardigrade proteins
Source: Protein Sci. 2024 Aug 16;33(9):e5135. doi: 10.1002/pro.5135 (PMC11328126; doi:10.1002/pro.5135)
Supplement: Supplementary file 1 — Data S1. Supporting information. [file PRO-33-e5135-s001.pdf]

ORIGINAL ARTICLE

Supplementary Information for: Structural adaptability and surface activity of tardigrade-inspired peptides

Giulia Giubertoni[1*] | Sarah Chagri[2*] | Pablo G. Argudo[2] | Leon Prädel[2] | Daria Maltseva[2] | Alessandro Greco[2] | Federico Caporaletti[1,4] | Alberto Pavan[1] | Ioana M.Ilie[1] | Yong Ren[2] | David Ng[2] | Mischa Bonn[2] | Tanja Weil[2] | Sander Woutersen[1]

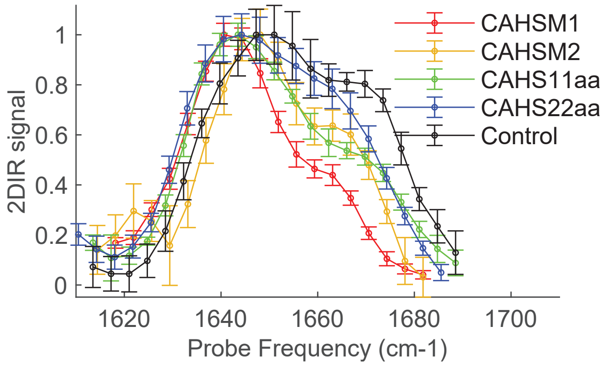

FIG. S1 Comparison between 2D-IR diagonal slices of the bleach signals of the isotropic 2D-IR spectra of the 5 different peptides.

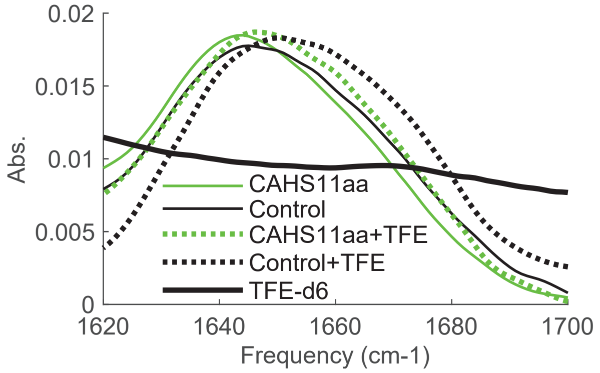

FIG. S2 Infrared spectra of CAHS11aa and control peptided at 0 and 50% TFE. We also report the IR spectrum of deuterated TFE (x 0.2), which also shows absorption bands in the amide I region. When subtracting the TFE IR spectrum to the peptide IR spectra, this overlap can cause problems leading to distortions in the background-corrected peptide spectra. These problems are overcome in 2D-IR since the TFE contribution to the 2D-IR spectrum is insignificant because 2D-IR signal scales as σ^2, and the σ of the TFE absorption band is much lower than the ones of the amide I modes.

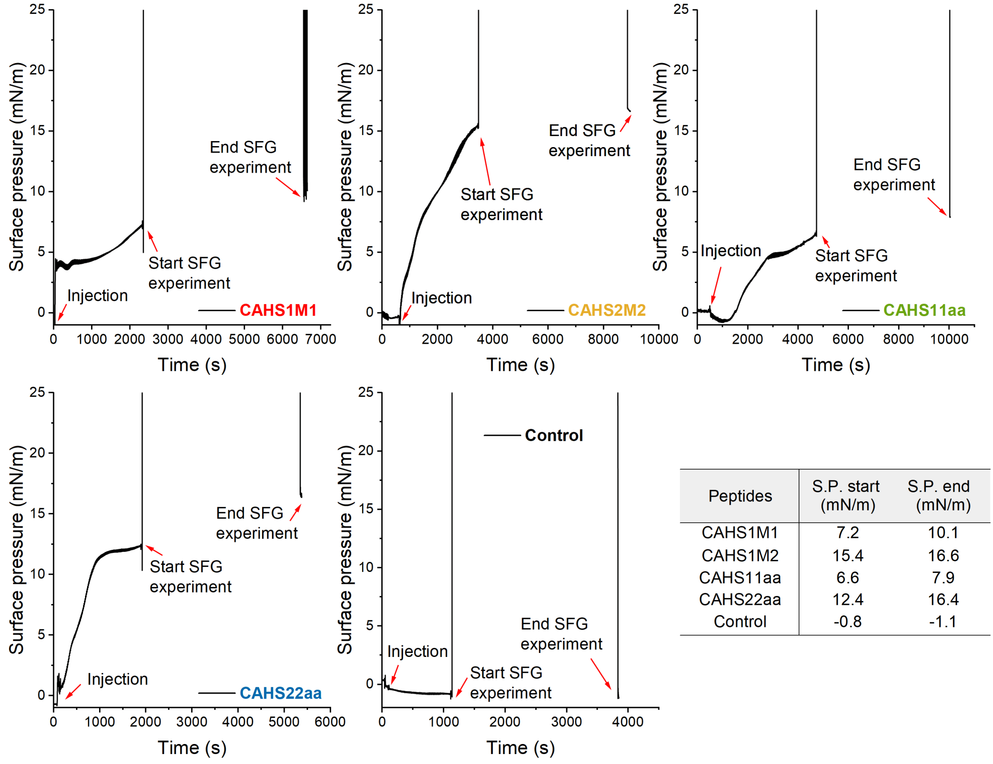

Peptides	S.P. start (mN/m)	S.P. end (mN/m)
CAHS1M1	7.2	10.1
CAHS1M2	15.4	16.6
CAHS11aa	6.6	7.9
CAHS22aa	12.4	16.4
Control	-0.8	-1.1

FIG. S3 Surface pressure of all peptides at 0.09 mg/ml in buffer solution and neutral pH. Note that the values were obtained before and after the SFG experiment was performed, which explains the missing values in this time range.

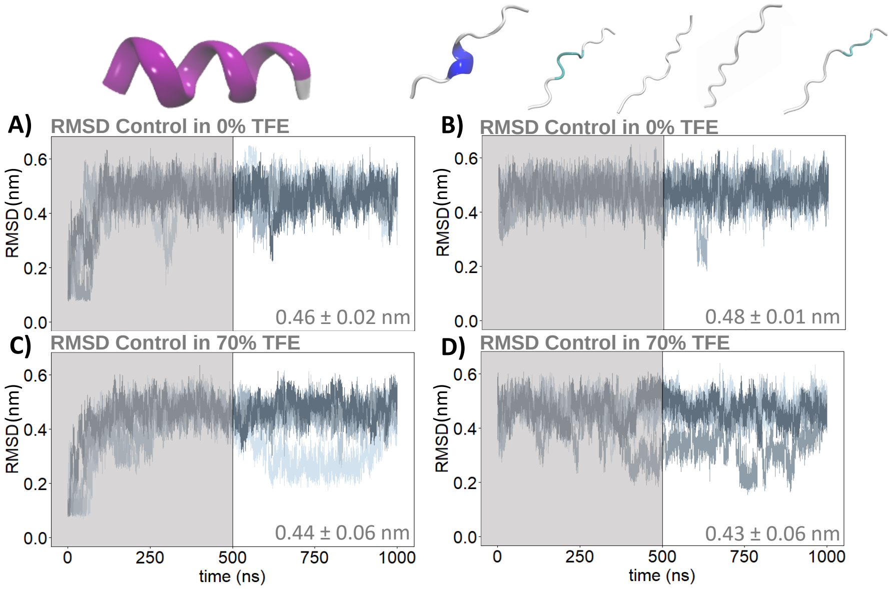

FIG. S4 Shown is the time series of the root mean square deviations of the control peptides from the extended helical conformation predicted by Alphafold2 [1]. Two sets of simulations are carried out for each system, *i.e.* starting from helical conformations (A,C) and from randomly generated conformations (B,D). First the structural alignment of the individual snapshots saved along the MD simulations is carried out on the C_α atoms of the peptides. Then for each MD snapshot the antibody C_α root-mean-square deviation (RMSD) is calculated as $\sqrt{\frac{1}{N_{ab}}\sum_{i=1}^{N_{ab}}(\mathbf{r_i}-\mathbf{r_i^{ref}})^2}$, where $\mathbf{r_i}$ and $\mathbf{r_i^{ref}}$ are the actual and reference coordinates, respectively. N_{ab} is the number of residues in the antibody variable domain. The first 500 ns of the simulations are discarded as equilibration. The values in the plots represent the average RMSD and the error bars represent the standard error of the mean calculated as the standard deviation of the average values over the independent runs.

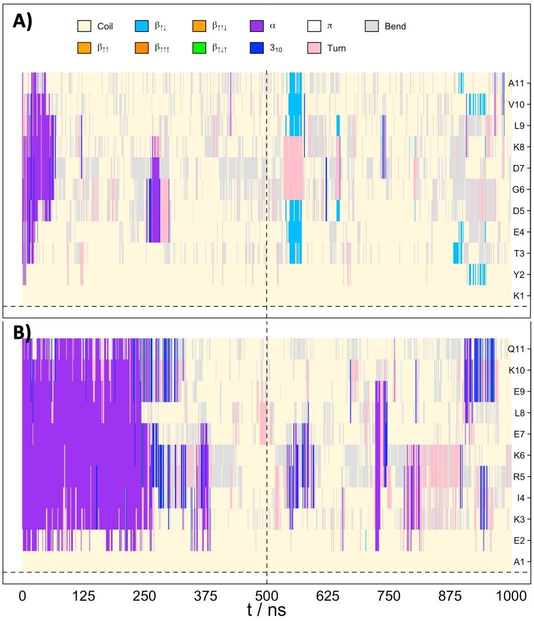

FIG. S5 Time series of the secondary structure per residue of the control peptide (A) and CAHS11aa (B) in a single run. The vertical dashed line indicates the separation between the equilibration and the production in the run.

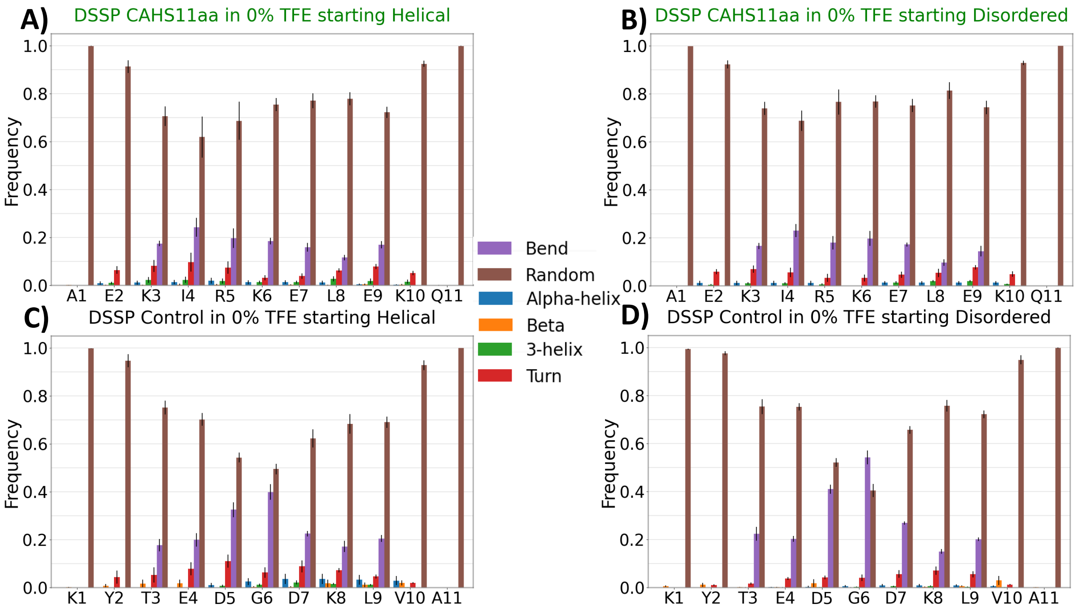

FIG. S6 Average secondary structure analysis per residue in CAHS11aa and control peptide in hydrating conditions. Two sets of molecular dynamics simulations were set up, from helical (A,C) and random (B,D) conformations for both peptides. The error bars represent the standard error of the mean calculated as the standard deviation of the average values over the independent runs.

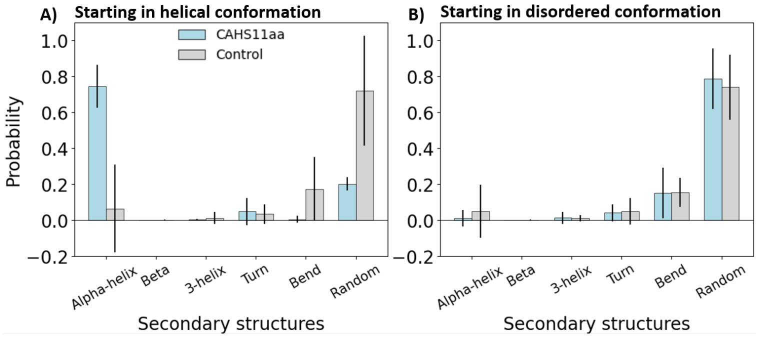

FIG. S7 Secondary structure analysis in 70 % TFE when averaged over the contributions of all residues. Two different sets of simulations are analysed: started from helical structures (A) and started from randomly generated conformations (B). The error bars represent the standard error of the mean calculated as the standard deviation of the average values over the independent runs.

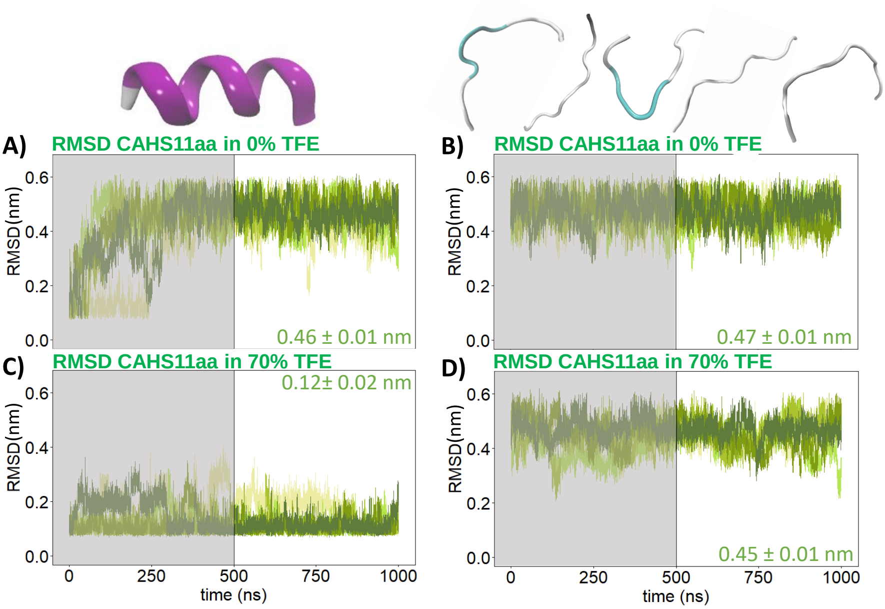

FIG. S8 Shown is the time series of the root mean square deviations of the peptides from the extended helical conformation modified from the CAHS11aa peptide through mutations. Two sets of simulations are carried out for each system, *i.e.* starting from helical conformations (A,C) and from randomly generated conformations (B,D). First the structural alignment of the individual snapshots saved along the MD simulations is carried out on the C_α atoms of the peptides. Then for each MD snapshot the antibody C_α root-mean-square deviation (RMSD) is calculated as $\sqrt{\frac{1}{N_{ab}}\sum_{i=1}^{N_{ab}}(\mathbf{r_i}-\mathbf{r_i^{ref}})^2}$, where $\mathbf{r_i}$ and $\mathbf{r_i^{ref}}$ are the actual and reference coordinates, respectively. N_{ab} is the number of residues in the antibody variable domain. The first 500 ns of the simulations are discarded as equilibration.

1 | METADYNAMICS ANALYSIS

To characterize the transitions between α and non-α structures, we calculated the free energy difference $\Delta G_{\alpha,1-\alpha}$ between the two states:

$$\exp\left[-\Delta G_{\alpha,1-\alpha}(\Delta S)/RT\right] = \frac{\iint_{\alpha} e^{-A(S_1,S_2)/RT} dS_1 dS_2}{\iint_{1-\alpha} e^{-A(S_1,S_2)/RT} dS_1 dS_2} \tag{1}$$

where S_1 and S_2 run from $N - \Delta S$ to N in the integrals over α along the free energy profile $A(S_1, S_2)$ and over all the non-α states in the integrals over $1-\alpha$. We extracted the optimal ΔS value, *i.e.* the deviations for which we sample conformations close to the initial extended helical conformation, from the $\Delta G_{\alpha,1-\alpha}$ dependence on ΔS (Fig. 9). For simplicity, we chose square regions in the conformation space, with $S_1 = S_2 = \Delta S$. Briefly, the profiles rapidly decreases with increasing ΔS values, *i.e.* with less stringent boundaries on the consideration of α helical structures. The decay levels off for CAHS11aa beyond $\Delta S = 1.5$. A less evident plateau is observed for the control peptide, which further supports the findings that the non-α structure is not stable in solution.

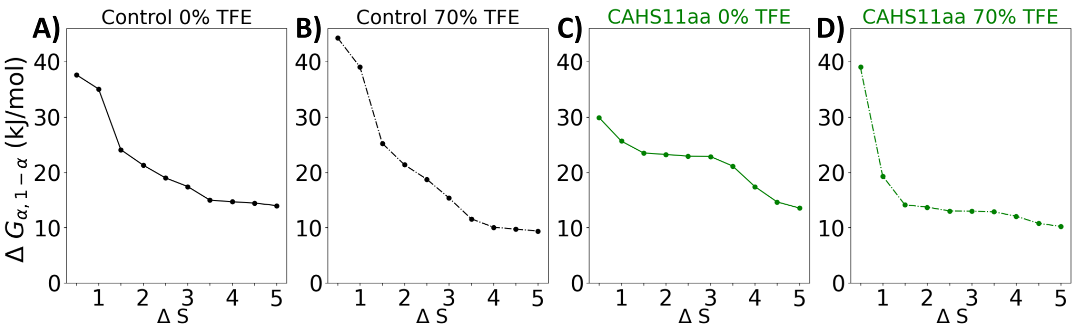

FIG. S9 Free energy difference $\Delta G_{\alpha,1-\alpha}(\Delta S)$ between the α and non-α-structures as a function of ΔS calculated from eq. 1 for the peptides in hydrating and dessecating conditions. Larger values of ΔS correspond to bigger squares in the upper right corner of Fig. 4.

Table S1: Peak fitting parameters for SSP SFG spectra in Figure 4. A_{NR} and φ_{NR} are the amplitude and phase of the non-resonant signal, respectively. A_n is the amplitude of the resonant signal, ω_n is the resonant frequency, and Γ_n is the width of transition.

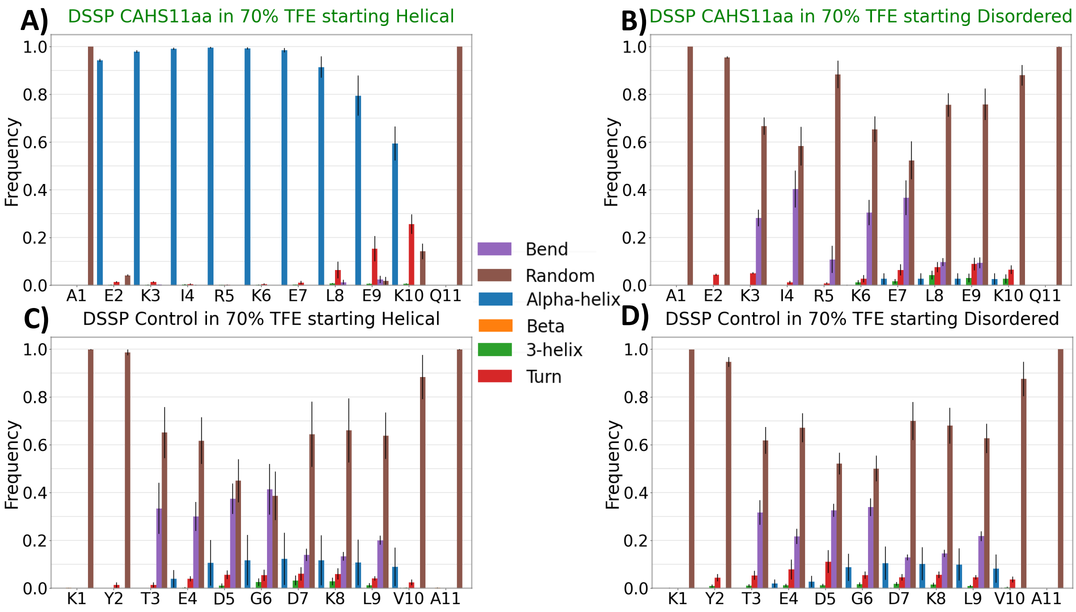

FIG. S10 Average secondary structure analysis per residue in CAHS11aa and control peptide in desiccating conditions. Two sets of molecular dynamics simulations were set up, from helical (A,C) and random (B,D) conformations for both peptides. The error bars represent the standard error of the mean calculated as the standard deviation of the average values over the independent runs.

references

[1] Jumper J, Evans R, Pritzel A, Green T, Figurnov M, Ronneberger O, et al. Highly accurate protein structure prediction with AlphaFold. Nature 2021;596(7873):583–589.

	CAHS1M1	CAHS1M2	CAHS11aa	CAHS22aa	Control	Buffer
A_{NR}	0.047					
φ_{NR}	1.385					
A (HOH bend)	-8.8					
ω (HOH bend)	1658					
Γ (HOH bend)	81.5					
ω_1(cm^{-1})	1642				–	
A_1	7.1	1.4	0.5	7.2	–	–
Γ_1 (cm^{-1})	39.2	32.6	22	26.7	–	–
ω_2(cm^{-1})	1653				–	
A_2	-0.3	7.6	6.9	-0.9	–	–
Γ_2 (cm^{-1})	11.5	52.4	42.9	12.1	–	–
ω_3 (cm^{-1})	1717				–	
A_3	-3.9	-3.5	-4.4	-4.4	–	–
Γ_3 (cm^{-1})	31.4	27.4	32.5	38.1	–	–
